# Phytocompounds vs. Dental Plaque Bacteria: *In vitro* Effects of Myrtle and Pomegranate Polyphenolic Extracts Against Single-Species and Multispecies Oral Biofilms

**DOI:** 10.3389/fmicb.2020.592265

**Published:** 2020-11-05

**Authors:** Daniela Sateriale, Roberta Imperatore, Roberta Colicchio, Chiara Pagliuca, Ettore Varricchio, Maria Grazia Volpe, Paola Salvatore, Marina Paolucci, Caterina Pagliarulo

**Affiliations:** ^1^Department of Science and Technology, University of Sannio, Benevento, Italy; ^2^Department of Molecular Medicine and Medical Biotechnology, Federico II University, Naples, Italy; ^3^Institute of Food Science-National Research Council, Avellino, Italy; ^4^CEINGE, Advanced Biotechnologies s.c.ar.l., Naples, Italy

**Keywords:** *in vitro* antibiofilm agents, polyphenolic extracts, dental plaque bacteria, *Streptococcus mutans*, *Streptococcus oralis*, *Streptococcus mitis*, *Rothia dentocariosa*

## Abstract

In the last decades, resistant microbial infection rate has dramatically increased, especially infections due to biofilm-producing strains that require increasingly complex treatments and are responsible for the increased mortality percentages compared with other infectious diseases. Considering that biofilms represent a key factor for a wide range of chronic infections with high drug tolerance, the treatment of biofilm-causing bacterial infections represents a great challenge for the future. Among new alternative strategies to conventional antimicrobial agents, the scientific interest has shifted to the study of biologically active compounds from plant-related extracts with known antimicrobial properties, in order to also evaluate their antibiofilm activity. In this regard, the aim of this study has been to assess the antibiofilm activity of polyphenolic extracts from myrtle leaf and pomegranate peel against oral pathogens of dental plaque, an excellent polymicrobial biofilm model. In particular, the *in vitro* antibiofilm properties of myrtle and pomegranate extracts, also in binary combination, were highlighted. In addition to inhibiting the biofilm formation, the tested polyphenolic extracts have been proven to destroy both preformed single-species and multispecies biofilms formed by *Streptococcus mutans*, *Streptococcus oralis*, *Streptococcus mitis*, and *Rothia dentocariosa* oral isolates, suggesting that the new natural sources are rich in promising compounds able to counteract biofilm-related infections.

## Introduction

Biofilms are complex microbial aggregations encapsulated in a hydrated polymeric matrix of their own synthesis (Aparna and Yadav, 2008). This bacterial complex limits the penetration of antimicrobial drugs, thus protecting the microbial cells entrapped. Furthermore, the biofilm microenvironment is favorable to microbial proliferation and exchange of genetic material, including the transmission of resistance genes (Chambless et al., 2006). About 80% of human bacterial infections were shown to be caused by biofilm-associated microorganisms (Wenzel, 2007), and more than half of infectious diseases related to biofilm formation involves bacterial species that are commensal or common in the human body environment (WHO, 2014). Oral bacteria are an example of human opportunistic species that can cause biofilm-related infections. Epithelial cells, dental surfaces, and orthodontic prosthesis are some of the numerous surfaces prone to the establishment of multispecies biofilms in the oral cavity (Marsh, 2004). They can cause several infectious diseases, including gingivitis, periodontitis, and dental caries, that still represent some of the most common chronic diseases, in both children and adults (Nishikawara et al., 2007). In addition, some oral bacterial species can also be responsible for critical infections outside the oral environment, such as bacteremia (Yang et al., 2009), endocarditis (Keng et al., 2012), and peritonitis (Khan et al., 2014). The oral microbiota can act as a reservoir for respiratory pathogens. Therefore, there is a close association between oral bacterial species and several forms of lung diseases, such as pneumonia (Raghavendran et al., 2007). *Streptococcus mutans* is considered the most cariogenic of all oral streptococci but is also implicated in subacute bacterial endocarditis and other extraoral pathologies, like nephropathy and atherosclerosis (Lemos et al., 2019). *Streptococcus oralis* belongs to the human oral microbiota but is also capable of opportunistic infections. It has been related to periodontal diseases and also to bacterial endocarditis (Byers et al., 2000), otitis media, septicemia, and pneumonia in children, in association with *Streptococcus pneumoniae* (Whalan et al., 2006). *Streptococcus mitis* and *Rothia dentocariosa* are commensal bacteria of the human mouth and the upper respiratory tract. These usually represent the etiologic agents in odontogenic infections and may contribute to dental caries. In addition, outside their niche, they can cause a wide range of infectious complications, such as endocarditis and septicemia (Mitchell, 2011).

It has been reported that bacteria living within biofilm, also in oral ones, are more tolerant to antibiotics as they are insensitive to the host’s immune response (Caraher et al., 2007). Therefore, biofilm-related infections can persist for a long time, thus progressing from acute infections to chronic infections (Leid et al., 2002). The increasing critical role of biofilms in pathogenesis and antimicrobial resistance led scientists to consider this complex structure as a drug target to tackle resistant infections. Unconventional antibiofilm agents with demonstrated antimicrobial activity could represent important alternatives for drug development to counteract infections due to major biofilm-forming pathogens.

In the last decades, the natural compounds have attracted the attention of scientists (Cowan, 1999). Since plant extracts have been used for the treatment of various infectious diseases for hundreds of years, they have sparked considerable interest in this context (Ahmad and Beg, 2001). Technological advances in modern science have accelerated the discovery of new phytocompounds of plant origin with therapeutic activity and without side effects (Ahmad and Aqil, 2009). The role of several phytocompounds, like polyphenols, as anti-infective agents is well-established today (Gibbons, 2005; Savoia, 2012). Among constituents of plants, polyphenols have received great scientific interest due to their numerous biological functions, such as promising activity against bacterial and fungal infections (Slobodníková et al., 2016). However, research regarding the antimicrobial effects of active plant-derived constituents against resistant biofilms appears still incomplete, compared to the demonstrated effects against their planktonic counterparts (Costerton et al., 2003).

Among the extensively studied natural sources rich in phytocompounds, especially polyphenols, *Myrtus communis* L. and *Punica granatum* L. could represent safe and economical alternatives to antibiotics, in the struggle against resistant infections caused by biofilm-related microorganisms. The antibacterial and antifungal effects of myrtle extracts (*M. communis* L.) were the object of recent scientific investigations. Crude extracts and essential oils of *M. communis* seem to be rich in polyphenols and terpenoids (Ben Hsouna et al., 2014), natural constituents of several plant portions that mediate a remarkable antioxidant activity along with strong antimicrobial effects against several pathogens (Aleksic and Knezevic, 2014). A wide plethora of scientific studies have also shown the significant properties of peel and juice pomegranate (*P. granatum* L.) extracts, in particular their anti-inflammatory and antimicrobial effects derived from the high content of polyphenols, mainly including ellagitannins and anthocyanins (Ismail et al., 2012; Pagliarulo et al., 2016; Ferrazzano et al., 2017). Most of the studies have investigated the antimicrobial activity of natural extracts against planktonic forms. In comparison, the experimental evidences of their antibiofilm effects are still few. Furthermore, the current antibiotic therapies showed very limited effectiveness to contrast the biofilm infection (Aparna and Yadav, 2008). Given the requirement to discover novel agents, preferably natural, with antibiofilm activity, as well as activity against oral biofilms, the aim of this study has been to define the antibiofilm profile of characterized polyphenolic extracts derived from myrtle leaf and pomegranate peel against *S. mutans*, *S. oralis*, *S. mitis*, and *R. dentocariosa* clinical isolates, important representative members of the dental plaque. Dental plaque bacteria are among the main microorganisms causing biofilm-related infections (from dental caries to systemic diseases). In particular, several studies have revealed that *S. mutans* represents about the 20–40% of the cultivable flora in biofilms removed from carious lesion (Rosenbloom and Tinanoff, 1991; Koo et al., 2013) and that *S. oralis* is a cariogenic bacterium significantly concurring in dental plaque formation (Jung et al., 2016). Recent reports also suggest that *S. mitis* and *R. dentocariosa* are common inhabitants of biofilms in the oral cavity that could be opportunistic pathogens, causing dental caries and periodontal pathologies (Peros et al., 2011; Banas et al., 2016). These reasons led us to consider the selected pathogenic bacterial strains as good candidates for the study of *in vitro* biofilm models useful in identifying new antibiofilm natural agents. The development of new knowledge about biofilm interferers/inhibitors could highlight novel, effective, and low-cost antibiofilm compounds that may find useful medical and environmental applications in the future.

## Materials and Methods

### Polyphenolic Extracts

The hydroethanolic extracts tested in this study were prepared with a solid–liquid solvent-extraction method from samples of dry myrtle leaf and dry pomegranate fruit peel, harvested from plants growing in the southern Italy countryside in the Salerno and Avellino areas, respectively. In particular, the preparation procedure of myrtle (*M. communis* L.) polyphenolic extract is described by Sateriale et al. (2020). Briefly, the myrtle leaf powder was mixed with a solution of ethanol:water (50:50), reaching a final concentration of 0.1 g ml^–1^. The suspension was stirred at room temperature (RT) for 30 min, using a rotary shaker, and centrifuged at 10,000 rpm for 15 min at RT. Then the supernatant was filtered through a single-use vacuum filtration unit (Sterilcup^®^/Steriltop^®^ Filtration System, Merck-Millipore, Darmstadt, Germany) with a 0.45 μm porosity membrane, by using a water vacuum pump. The polyphenolic extract of *P. granatum* L. fruit peel was prepared according to Pagliarulo et al. (2016), with some minor changes. Pomegranate peel powder (5 g) was homogenized in 25 ml of ethanol:water (50:50) solution for 30 min at RT in the dark. After centrifugation (10,000 rpm for 15 min at RT), the supernatant was filtered by the vacuum filtration systems (0.45 μm porosity membrane) described for myrtle extracts.

Extract volume was reduced by a rotavapor (Heidolph 36001270 Hei-VAP Precision Rotary Evaporator) and finally lyophilized. The resuspension of lyophilized polyphenols in fresh solvent was carried out before each antimicrobial test, adjusting the concentration according to the requirements of the performed assays.

### Bacterial Isolates and Growth Conditions

The antibiofilm activity of polyphenolic extracts was assessed against the *S. mutans* ATCC 25175 (LGC Standards, United Kingdom) strain, isolated from carious dentin, and against the clinical isolates of *S. oralis* SO1, *S. mitis* SM2, and *R. dentocariosa* RD1, obtained from samples of dental plaque from children with tooth decay, provided by the Pediatric Dentistry Department of “Federico II” University, Naples, Italy. Permission to take dental plaque samples was acquired according to the local planning authorities. Furthermore, approval for this study was granted by the ethics committee of the “Federico II” University, Naples, Italy (protocol number 101/14). The isolation of bacterial strains was carried out by culture techniques. The use of selective growth media allowed us to isolate representative colonies from dental plaque samples. The detailed identification of isolates was subsequently performed by mass spectrometry using the matrix-assisted laser desorption/ionization (MALDI) mass spectrometer (Bruker Daltonics, MALDI Biotyper, Fremont, CA, United States), a high-throughput proteomic technique for identification of a variety of bacterial species (Neville et al., 2011; Sogawa et al., 2011), and by a biochemical phenotyping method in a BD Phoenix Automated Microbiology System (Becton Dickinson, BD Franklin Lakes, NJ, United States), according to the manufacturer’s instruction.

Oral bacterial isolates were aerobically cultured at 37°C in brain heart infusion (BHI) agar/broth (Condalab, Torrejón de Ardoz, Madrid, Spain) and Columbia CNA agar base with 5% sheep blood and with colistin and nalidixic acid (Condalab, Torrejón de Ardoz, Madrid, Spain).

Bacterial isolates were kept in agar media at 4°C and were stored frozen at −80°C in BHI broth with 10% glycerol (v/v) (Carlo Erba, Reagents, Milan, Italy). Working cultures were activated in broth medium at 37°C for 15–18 h before use.

### Biofilm *in vitro* Reproduction

#### Tube Method

A qualitative and semiquantitative evaluation of biofilm formation was carried out, adapting the “tube method” (TM) described by Christensen et al. (1985) and Ansari et al. (2014). In brief, the bacterial isolates were subcultured in 5 ml of BHI broth tubes, by adjusting turbidity to an optical density (OD) of 0.5 at A_600*nm*_, and aerobically incubated for 48 h at 37°C. Subsequently, the medium was discarded, and the tubes were washed with phosphate buffer saline (1xPBS, pH 7.3) and dried. The bacterial cells adherent to the tubes were then stained with crystal violet (2%) for 1 min. After removal of the stain excess, tubes were washed with deionized water, dried in an inverted position, and observed for biofilm formation. The isolates were considered as positive when a visible biofilm lined the wall and bottom of the tube, while a simple ring formation at the liquid interface was not indicative of biofilm formation. Based on the careful observation by the operator, the microbial isolates have been classified as negative (0), when no visible biofilm was observed; weakly positive (+), when a visible biofilm was observed; moderately positive (+ +), when a twice darker visible biofilm was observed with respect to weakly positive cases; and strongly positive (+ + +), when a three times darker visible biofilm was observed with respect to weakly positive cases. Each microorganism suspension at 0.5 OD_600 *nm*_ was inoculated in a ratio of 1:1:1:1 in mixed culture to form a multispecies biofilm.

#### Tissue Culture Plate Method

The biofilm biomass measurement of selected oral isolates was evaluated by the quantitative assay known as “tissue culture plate method” (TCPM), similar to that described by Costa et al. (2013). BHI broth (10 ml) was inoculated with test microorganisms from overnight cultures at 37°C for 24 h. After aerobic incubation, each fresh culture was further adjusted by reaching the OD at 0.5, by spectrophotometric reading at A_600 *nm*_, and aliquots of 0.2 ml of starter culture were dispensed, in six replicates, into each well of a 96-well flat-bottomed microplate for tissue cultures in hydrophilic polystyrene (Nunc^TM^ MicroWell 96-well microplates, Thermo Scientific, Denmark). Each microorganism suspension at 0.5 OD_600 *nm*_ was inoculated in a ratio of 1:4 with others to form a multispecies biofilm. Sterile BHI broth was used as negative control. The culture plates were incubated at 37°C for 3–6–9–12–18–24–36–48 h, without shaking. Different plates were used for different incubation times. For incubation times longer than 3 h, non-adherent bacteria were removed at regular intervals (T_3_–T_6_–T_9_–T_12_–T_18_–T_24_–T_36_–T_48_), new fresh BHI broth medium was added, and plates were further incubated for the remaining time, according to the established total incubation period. After each incubation, the bacterial culture was removed, and the wells were washed with 0.2 ml of 1xPBS (pH 7.3) three times to remove free-floating bacteria. The biofilms adherent to the wells were fixed with 0.2 ml of 85% ethanol (Sigma-Aldrich, Merck KGaA, Darmstadt, Germany) for 15 min and stained with 0.2% crystal violet (Sigma-Aldrich, Merck KGaA, Darmstadt, Germany) for 5 min. The stain excess was washed with deionized water, and plates were dried in a thermostat at a temperature of 30°C for 10 min upside down. The OD of the purple-stained solution was recorded at 600 nm wavelength by a microplate reader (Bio-Rad microplate reader, Model 680). Based on the measurement of OD (OD_*I*_) compared to the OD of sterile BHI broth used as negative control (OD_*C*_), the microbial isolates have been classified as non-adherent (OD_*I*_ ≤ OD_*C*_), weakly adherent (OD_*C*_ < OD_*I*_ ≤ 2^∗^OD_*C*_), moderately adherent (2^∗^OD_*C*_ < OD_*I*_ ≤ 4^∗^OD_*C*_), and strongly adherent (4^∗^OD_*C*_ < OD_*I*_).

### *In vitro* Antibiofilm Assays With Polyphenolic Extracts

#### Biofilm Formation Inhibition Assay

To evaluate the ability of the myrtle and pomegranate hydroethanolic polyphenolic extracts to inhibit biofilm formation, some modifications to the tissue culture plate method have been proposed. In particular, a volume of 0.1 ml of extracts, at sub-MIC (1/4 MIC and 1/2 MIC), MIC, and over-MIC (2 MIC and 4 MIC) concentrations, was added to each well of a 96-well microplate. The same volume of BHI broth was added to replace the extracts in the negative control. Finally, 0.1 ml of each single and mixed bacterial culture, with 0.5 OD at A_600 *nm*_ wavelength, was pipetted to each well, reaching a final volume of 0.2 ml. BHI (0.2 ml) broth was added in wells without bacterial culture as a blank. The plates were wrapped loosely and aerobically incubated at 37°C for 24 h without shaking to allow the cells to attach to the surface. After the incubation, the content of each well was removed, and all the subsequent steps of the above-mentioned TCPM have been performed. After staining with crystal violet, reading at A_600 *nm*_ by a microplate reader was performed. Results were given as a percentage of biofilm formation inhibition, applying the following formula, according to Bakkiyaraj et al. (2013):

$$\mathrm{Biofilm}\mathrm{formation}\mathrm{inhibition}\%={\left(\frac{{\mathrm{OD}}_{\mathrm{control}}-{\mathrm{OD}}_{\mathrm{assay}}}{{\mathrm{OD}}_{\mathrm{control}}}\right)}^{*}100$$

where *OD*_*control*_ is the mean OD measured for bacterial biofilms grown without extracts, while *OD*_*assay*_ is the mean OD measured for bacterial biofilms grown in the presence of myrtle and pomegranate hydroalcoholic polyphenolic extracts, as single agents and in binary combination.

The lowest concentration of each extract, or extract binary combination, that produced biofilm inhibition was considered to be the minimum biofilm inhibition concentration (MBIC).

#### Biofilm Eradication Assay

To assess the effects of myrtle and pomegranate hydroethanolic polyphenolic extracts upon mature 1-day biofilms, some adaptations of the TCPM biofilm formation protocol were performed. In particular, aliquots of 0.2 ml of single/mixed starter bacterial culture (with adjusted OD at 0.5 OD_600 *nm*_) were dispensed, in six replicates, into each well of a 96-well flat-bottomed microplate. BHI broth (0.2 ml) was added into wells without bacterial culture as a blank. The plates were wrapped loosely and aerobically incubated at 37°C for 24 h without shaking, to allow the cells to attach to the surface. Following incubation, the content of each well was removed, and a volume of 0.1 ml of extracts was added, at MBIC and over-MBIC (2 MBIC, 3 MBIC, and 4 MBIC) concentrations. The same volume of BHI broth was added as a negative control. Finally, 0.1 ml of fresh BHI broth was added to each well, reaching a final volume of 0.2 ml. Fresh BHI broth (0.2 ml) was added in wells without bacteria culture as a blank. The plates were again aerobically incubated at 37°C for 24 h. After the second incubation, the washings with PBS, the staining with 0.2% crystal violet, and the final washing with distilled water were performed before reading the plates at A_600 *nm*_ wavelength. Results for this test were given as a percentage of biofilm disruption, calculated by the following formula, according to Bakkiyaraj et al. (2013):

$$Biofilmdisruption\%={\left(\frac{OD_{control}-OD_{assay}}{OD_{control}}\right)}^{*}100$$

where *OD*_*control*_ is the mean OD measured for 1-day bacterial biofilms without extracts, while *OD*_*assay*_ is the mean OD measured for 1-day bacterial biofilms treated with myrtle and pomegranate hydroalcoholic polyphenolic extracts, as single agents and in binary combination.

The lowest concentration of each extract, or binary combination of extracts, that is able to destroy (eradicate) preformed biofilms was considered to be the minimum biofilm eradication concentration (MBEC).

#### Biofilm Visualization Through Microscopy Techniques

##### Light microscopy analysis

The ability of myrtle and pomegranate polyphenolic hydroalcoholic extracts (50% v/v) in binary combination to inhibit biofilm formation has been confirmed by a microscopic technique similar to that described by Chaieb et al. (2011), with minor modifications. Briefly, the biofilm of each separate bacterial strain and also that of the multispecies biofilm formed by *S. mutans*, *S. oralis*, *S. mitis*, and *R. dentocariosa* were grown on glass cover slides (1 cm^2^) placed in 24-well polystyrene plates (Nunc^TM^ MicroWell 96-well microplates, Thermo Fisher Scientific, Denmark). In particular, aliquots of 0.2 ml of single/mixed starter bacterial culture (with an adjusted OD at 0.5 OD_600*nm*_) were dispensed on the cover glass, and then the wells were filled with different concentrations of extracts (0, 20, 40 μg μl^–1^), reaching a final volume of 0.4 ml in each well, before aerobic incubation at 37°C for 24 h. For negative control, bacterial cultures without extracts were used. After incubation, wells were emptied, washed with PBS, fixed with 90% ethanol for 15 min, and completely dried at 30°C. Then biofilms were stained with 1% crystal violet (Sigma-Aldrich, Merck KGaA, Darmstadt, Germany) for 20 min at RT. The excess dye was washed with distilled water. Finally, dried stained glass pieces were placed on slides and were observed with a trinocular light microscope (Motic B1 Series, Model B1-223 A) at 40X magnification with 40X/0.65/S (WD 0.53 mm) objective.

##### Fluorescence microscopy analysis

Biofilm architecture, in the absence and in the presence of myrtle and pomegranate hydroalcoholic extracts in binary combination, was evaluated by fluorescence microscopy analysis. The performed method, adapted from Singh et al. (2012), consisted in assessing single and polymicrobial biofilms on glass coverslips (1 cm^2^) immersed in bacterial culture (0.5 OD_600 *nm*_) in 24-well plates. In particular, aliquots of 0.2 ml of single/mixed starter bacterial cultures were dispensed on the cover glass, and then the wells were filled with different concentrations of extracts (0, 20, and 40 μg μl^–1^), reaching a final volume of 0.4 ml in each well, before. Bacterial cultures without extracts were used as negative control. After aerobic incubation at 37°C for 24 h, mature-biofilm wells were emptied, washed with PBS, fixed with 90% ethanol for 15 min, and completely dried at 30°C. Then biofilms were stained with 1 mM propidium iodide (PI; product code 81845, Sigma-Aldrich, Merck KGaA, Darmstadt, Germany) for 15 min at RT. The excess of dye was washed with distilled water. Finally, biofilms were observed with a fluorescent microscope Nikon Eclipse Ti-S (Nikon, Florence, Italy) equipped with a digital camera DS-Qi2 (Nikon, Florence, Italy) and the acquisition and image analysis software NIS-Elements C (Nikon, Florence, Italy). Digital images were acquired using the 40X objective. PI (excitation wavelength, 543 nm; fluorescence emission wavelength, 617 nm) emits red fluorescence. All fluorescence images were analyzed by Fiji ImageJ software (National Institutes of Health, Bethesda, MD) to obtain the mean fluorescence intensities from biofilms.

### Statistical Data Analysis

All experiments were performed in triplicate, with independent microbial cultures for antimicrobial assays. The results obtained were analyzed and graphically reported by using GraphPad Prism 6 software, validating the statistical significance by the one-way ANOVA test with Tukey correction and the two-way ANOVA test with Bonferroni and Dunnett corrections. In all cases, *p* < 0.05 were considered statistically significant.

## Results

### Biofilm Production by *S. mutans* ATCC 25175, *S. oralis* SO1, *S. mitis* SM2, and *R. dentocariosa* RD1 Oral Isolates

**FIGURE 1:**
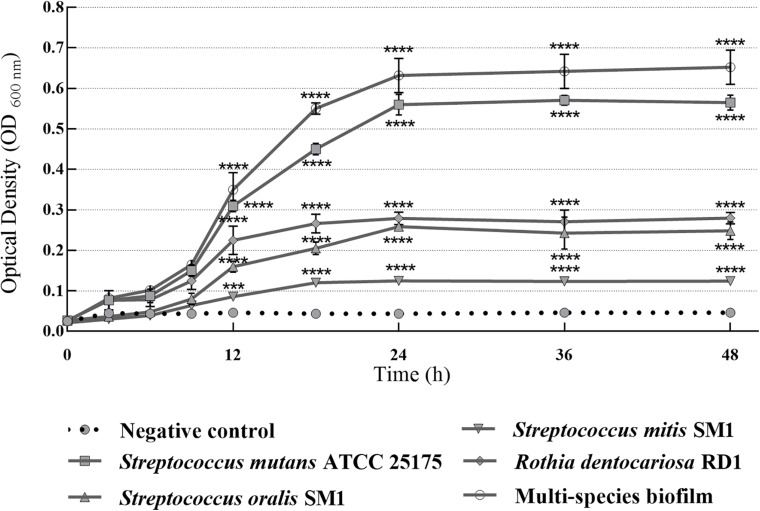
Absorbance values of crystal violet assay with single-species and multispecies biofilms (1:1:1:1 ratio for each isolate) produced by *S. mutans* ATCC 25175, *S. oralis* SO1, *S. mitis* SM2 and *R. dentocariosa* RD1 oral isolates, plotted over time. OD_600_ readings were taken after 3, 6, 9, 12, 18, 24, 36, and 48 h. The experiments were performed in triplicate with independent cultures, and statistical significance was examined by the two-way ANOVA test with Bonferroni correction. Results are indicated as means ± SDs. Asterisks indicate statistical significance (***p < 0.001; ****p < 0.0001).

In this study, *S. mutans* ATCC 25175, *S. oralis* SO1, *S. mitis* SM2, and *R. dentocariosa* RD1 biofilm-forming potential was qualitatively and quantitatively analyzed using the TM and the TCPM. The assigned adherence levels are shown in Table 1. A multispecies biofilm with the mentioned oral isolates was also set up and screened. To quantify the differences between the biofilm-forming ability of oral isolates over time, biofilm formation was monitored using crystal violet staining with regular intervals. Figure 1 shows the microplate measurements generated after static incubations at 37°C from 3 to 48 h (T_3_–T_6_–T_9_–T_12_–T_18_–T_24_–T_36_–T_48_).

**TABLE 1:** Biofilm adherence levels of *S. mutans* ATCC 25175, *S. oralis* SO1, *S. mitis* SM2, and *R. dentocariosa* RD1 oral isolates, in pure and mixed cultures.

Bacterial isolate	Mean control OD_600 *nm*_	Mean bacterial isolate OD_600 *nm*_	Bacterial adherence level
*S. mutans* ATCC 25175	0.057 ± 0.005	0.506 ± 0.045	Strongly adherent
*S. oralis* SO1	0.057 ± 0.005	0.224 ± 0.022	Moderately adherent
*S. mitis* SM2	0.057 ± 0.005	0.111 ± 0.011	Weakly adherent
*R. dentocariosa* RD1	0.057 ± 0.005	0.227 ± 0.020	Moderately adherent
Mixed culture (1:1:1:1 ratio)	0.057 ± 0.005	0.607 ± 0.028	Strongly adherent

*OD_600 nm_, optical density detected at a wavelength of 600 nm by a microplate reader.*

### *In vitro* Antibiofilm Activity of Myrtle Leaf and Pomegranate Peel Polyphenolic Extracts, as Single Agents and in Binary Combination, Against *S. mutans* ATCC 25175, *S. oralis* SO1, *S. mitis* SM2, and *R. dentocariosa* RD1 Oral Isolates

The antibiofilm activity of polyphenolic hydroethanolic extracts of myrtle leaf (MLE) and pomegranate peel (PPE), also in binary combination (MLE + PPE, 1:1 ratio), was measured against *S. mutans* ATCC 25175, *S. oralis* SO1, *S. mitis* SM2, and *R. dentocariosa* RD1 oral isolates at several concentrations, including sub-MIC (1/4 MIC and 1/2 MIC), MIC, and over-MIC (2 MIC and 4 MIC) concentrations, in order to assess the impact of the natural extracts upon biofilm formation of dental plaque pathogens involved in caries disease etiology.

MBIC values, which ranged between 10 μg μl^–1^ (MLE + PPE vs. *S. oralis* SO1 and *S. mitis* SM2) and 40 μg μl^–1^ (MLE vs. *S. mutans* ATCC 25175 and *R. dentocariosa* RD1, together with PPE vs. *S. mutans* ATCC 25175, *S. mitis* SM2, and *R. dentocariosa* RD1) (Table 2), were assigned to the lowest concentrations of antimicrobial agents required to inhibit the formation of biofilms, while MBEC values, assigned to the lowest concentration of each antimicrobial agent that is able to destroy (eradicate) preformed biofilms, ranged between 40 μg μl^–1^ (MLE + PPE vs. *S. mutans* ATCC 25175, *S. oralis* SO1, and *S. mitis* SM2) and 120 μg μl^–1^ (MLE vs. *S. mutans* ATCC 25175 and *R. dentocariosa* RD1, together with PPE vs. *S. mutans* ATCC 25175, *S. mitis* SM2, and *R. dentocariosa* RD1) (Table 2).

**TABLE 2:** Quantitative evaluation of effects of myrtle and pomegranate polyphenolic extracts, used individually and in binary combinations, against *S. mutans* ATCC 25175, *S. oralis* SO1, *S. mitis* SM2, and *R. dentocariosa* RD1 biofilms.

Antibacterial agent	*S. mutans* ATCC 25175		*S. oralis* SO1		*S. mitis* SM2		*R. dentocariosa* RD1	
	MBIC (μg μl^–1^)	MBEC (μg μl^–1^)	MBIC (μg μl^–1^)	MBEC (μg μl^–1^)	MBIC (μg μl^–1^)	MBEC (μg μl^–1^)	MBIC (μg μl^–1^)	MBEC (μg μl^–1^)
MLE	40.00	120.00	20.00	40.00	20.00	40.00	40.00	120.00
PPE	40.00	120.00	20.00	80.00	40.00	120.00	40.00	120.00
MLE + PPE (1:1 ratio)	20.00	40.00	10.00	40.00	10.00	40.00	20.00	80.00

*MLE, myrtle leaf 50% ethanolic extracts; PPE, pomegranate peel 50% ethanolic extracts; MBIC, minimum biofilm inhibition concentration; MBEC, minimum biofilm eradication concentration.*

The ability of the MLE, PPE, and MLE + PPE binary combination to disrupt preformed biofilms of *S. mutans* ATCC 25175, *S. oralis* SO1, *S. mitis* SM2, and *R. dentocariosa* RD1 was tested at MBIC and over-MBIC concentrations (2 MBIC, 3 MBIC, and 4 MBIC).

**FIGURE 2:**
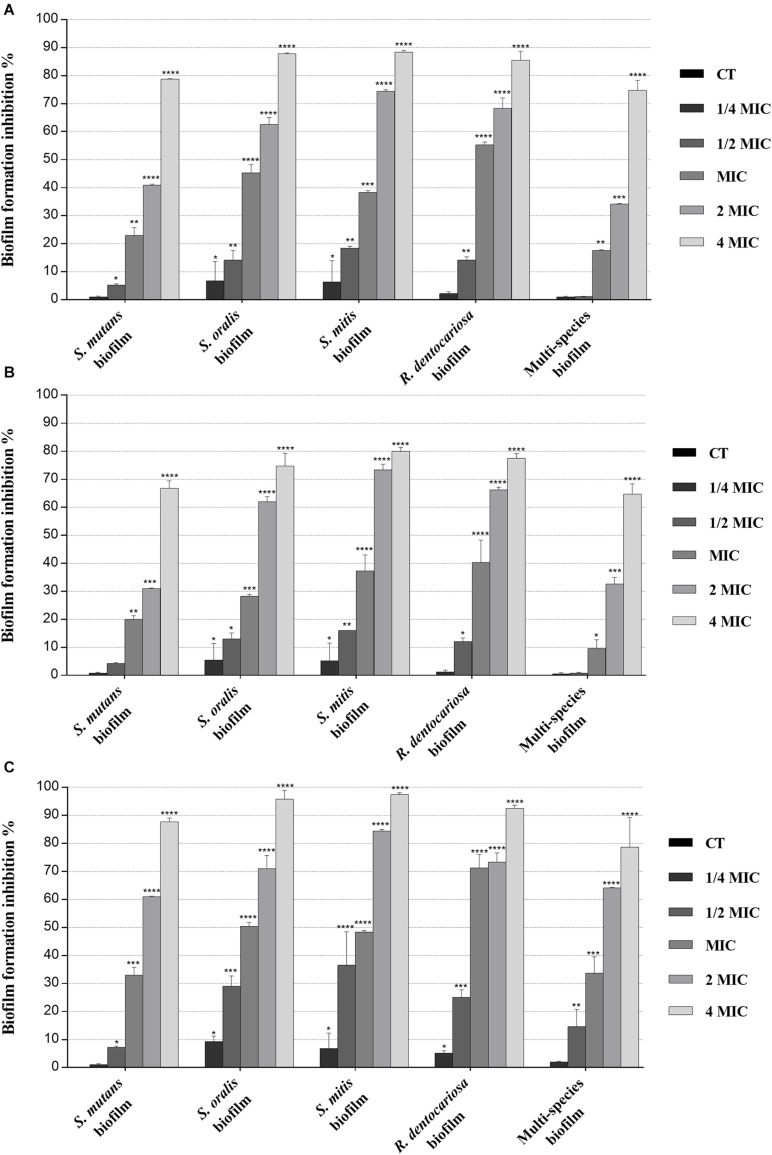
Inhibitory effect of myrtle and pomegranate polyphenolic extracts, used individually and in binary combinations, on single-species and multispecies biofilm formation by *S. mutans* ATCC 25175, *S. oralis* SO1, *S. mitis* SM2, and *R. dentocariosa* RD1 oral isolates. Figure shows the inhibition percentage values of *S. mutans* ATCC 25175, *S. oralis* SO1, *S. mitis* SM2, and *R. dentocariosa* RD1 in single-species and multispecies biofilm (1:1:1:1 ratio for each isolate) detected, in the presence of sub-MIC (1/4 MIC and 1/2 MIC), MIC, and over-MIC (2 and 4 MIC) concentrations of myrtle leaf 50% hydroethanolic extract (MLE) **(A)**; pomegranate peel 50% hydroethanolic extract (PPE) **(B)**; myrtle leaf 50% hydroethanolic extract in combination with pomegranate peel 50% hydroethanolic extract (MLE + PPE) added in a 1:1 ratio **(C)**. The experiments were performed in triplicate with independent cultures, and statistical significance was examined by the two-way ANOVA test with Dunnett correction. Results are indicated as means ± SDs. Asterisks indicate statistical significance (*p < 0.05; **p < 0.01;***p < 0.001; ****p < 0.0001).

**FIGURE 3:**
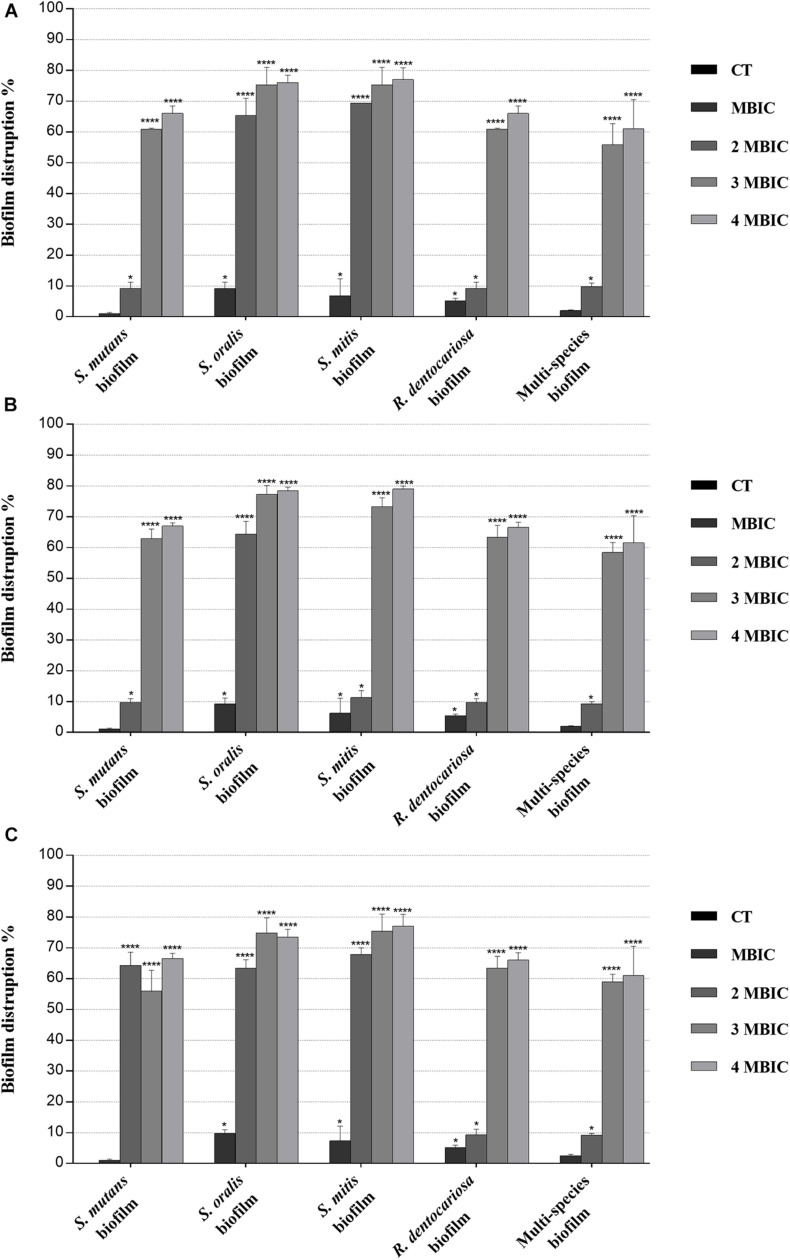
Biofilm eradication activity of myrtle and pomegranate polyphenolic extracts, also in binary combination, on single*-*species and multispecies mature biofilms of *S. mutans* ATCC 25175, *S. oralis* SO1, *S. mitis* SM2, and *R. dentocariosa* RD1 oral isolates. Figure shows the disruption percentage values of *S. mutans* ATCC 25175, *S. oralis* SO1, *S. mitis* SM2, and *R. dentocariosa* RD1 oral isolates in single and multispecies biofilms (1:1:1:1 ratio for each isolate) detected in the presence of MBIC and over-MBIC (2 MBIC, 3 MBIC, and 4 MBIC) concentrations of myrtle leaf 50% hydroethanolic extract (MLE) **(A)**; pomegranate peel 50% hydroethanolic extract (PPE) **(B)**; myrtle leaf 50% hydroethanolic extract in combination with pomegranate peel 50% hydroethanolic extract (MLE + PPE) added in a 1:1 ratio **(C)**. The experiments were performed in triplicate with independent cultures, and statistical significance was examined by the two-way ANOVA test with Dunnett correction. Results are indicated as means ± SDs. Asterisks indicate statistical significance (*p < 0.05; ****p < 0.0001).

Biofilm inhibition and eradication activity of myrtle and pomegranate polyphenolic extracts, as single agents and in binary combination, on single-species and multispecies mature biofilms of *S. mutans* ATCC 25175, *S. oralis* SO1, *S. mitis* SM2, and *R. dentocariosa* RD1 oral isolates are reported in Figures 2, 3, respectively.

Regarding the effect on biofilm formation, the hydroethanolic (50% v/v) polyphenolic extracts of *M. communis* L. leaf and *P. granatum* L. fruit peel produced a significant (*p* < 0.05) inhibition of *S. mutans* ATCC 25175, *S. oralis* SO1, *S. mitis* SM2, and *R. dentocariosa* RD1 biomass with respect to untreated control, as shown in Figure 2. In detail, both myrtle and pomegranate extracts, also in binary combination, at sub-MIC concentrations caused a slight or not significant decrease for all oral isolates, ranging from 1.2% (PPE vs. four-species biofilm) to 35.7% (MLE + PPE vs. *S. mitis* biofilm) with respect to the control (Figure 2), while both produced a significant (*p* < 0.05) inhibition of biofilm formation at MIC and over-MIC concentrations, with inhibition percentages reaching up to 97.3% for MLE + PPE binary combination against the *S. mitis* biofilm (Figure 2). The ability of the MLE, PPE, and MLE + PPE binary combination to disrupt preformed biofilms of *S. mutans* ATCC 25175, *S. oralis* SO1, *S. mitis* SM2, and *R. dentocariosa* RD1 was tested at MBIC and over-MBIC concentrations (2 MBIC, 3 MBIC, and 4 MBIC). It was found that preformed biofilms of all tested oral isolates were significantly disrupted at over-MBIC concentrations (Figure 3).

### Detection of *S. mutans* ATCC 25175, *S. oralis* SO1, *S. mitis* SM2, *R. dentocariosa* RD1, and Multispecies Biofilm Architecture in the Absence and in the Presence of Myrtle Leaf and Pomegranate Peel Polyphenolic Extracts

Microscopy techniques allowed us to visually confirm the effects of the polyphenolic extracts against single-species and multispecies biofilms produced by oral pathogens.

**FIGURE 4:**
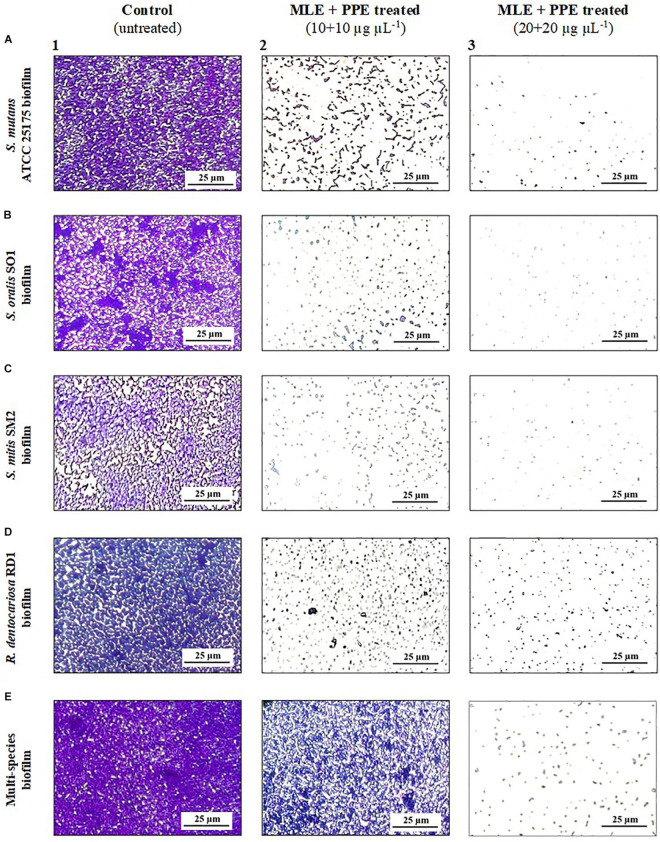
Light microscopy images of mature *S. mutans* ATCC 25175 **(A)**, *S. oralis* SO1 **(B)**, *S. mitis* SM2 **(C)**, *R. dentocariosa* RD1 **(D)**, and multispecies **(E)** biofilms (1:1:1:1 ratio for each isolate) grown with and without treatment with myrtle extract in binary combination with pomegranate extract. Crystal violet staining; MLE + PPE, myrtle leaf 50% ethanolic extracts in binary combination with pomegranate peel 50% ethanolic extracts (1:1 ratio).

In particular, light microscopy images (Figure 4) show the morphology of the mature biofilms (24 h) of *S. mutans* ATCC 25175, *S. oralis* SO1, *S. mitis* SM2, and *R. dentocariosa* RD1, in single species and multispecies, grown in the absence and in the presence of the binary combination of myrtle leaf 50% ethanolic extracts and pomegranate peel 50% ethanolic extracts (MLE + PPE). The images of single-species biofilm treated with MLE + PPE at increasing concentrations (Figures 4A2,A3,B2,B3,C2,C3,D2,D3) show a gradual reduction in biofilm mass at 20 and 40 μg μl^–1^ extract concentrations, compared to their respective untreated controls (Figures 4A1,B1,C1,D1). The extracts also demonstrated the ability to inhibit the multispecies biofilm formation with respect to the unexposed control (Figures 4E1–E3).

**FIGURE 5:**
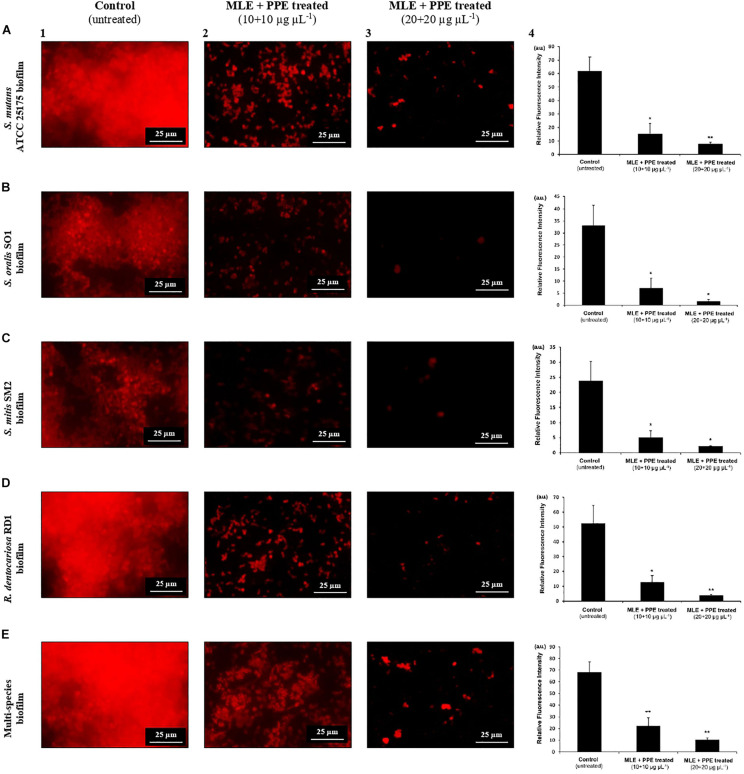
Fluorescence microscopy images of mature *S. mutans* ATCC 25175 **(A)**, *S. oralis* SO1 **(B)**, *S. mitis* SM2 **(C)**, *R. dentocariosa* RD1 **(D)**, and multispecies **(E)** biofilms (1:1:1:1 ratio for each isolate) grown with and without treatment with myrtle extract in binary combination with pomegranate extract. PI staining; MLE + PPE, myrtle leaf 50% ethanolic extract in binary combination with pomegranate peel 50% ethanolic extract (1:1 ratio). Relative fluorescence intensity of biofilm architecture in the *S. mutans* ATCC 25175 (A4), *S. oralis* SO1 (B4), *S. mitis* SM2 (C4), *R. dentocariosa* RD1 (D4) and multispecies (E4) biofilms is reported in arbitrary unit (a.u.). Data are expressed as mean ± SE. **p < 0.001,*p < 0.05 compared to the control group.

The effects of the binary combination of MLE and PPE on the architecture of biofilms formed by *S. mutans*, *S. oralis*, *S. mitis*, and *R. dentocariosa*, in single and mixed cultures, were analyzed also by fluorescence microscopy with PI-based fluorescent staining, as shown in Figure 5.

In fluorescence microscopy images, we can observe a large fraction of red PI-stained areas in single-species and multispecies biofilms on untreated glass (Figures 5A1,B1,C1,D1,E1), while the fractions of red-stained areas tended to decrease progressively on treated surfaces with the extracts at increasing concentrations with respect to untreated controls (Figures 5A2,A3,B2,B3,C2,C3,D2,D3,E2,E3). PI is a fluorescent intercalating agent widely used to stain microbial cells, also encapsulated in a biofilm matrix, and nucleic acids, including extracellular DNA (eDNA), which is one of the most abundant components of the exopolysaccharide matrix (Whitchurch et al., 2002). To confirm the decrease of red PI-stained areas in single-species and multispecies biofilms on the surfaces treated with the extracts, a quantitative analysis of the relative fluorescence intensity was performed. In particular, *S. mutans* single culture showed a significant decrease of relative fluorescence intensity in the presence of myrtle leaf and pomegranate peel polyphenolic extracts with respect to the control [control: 61.78 ± 10.57 vs. MLE + PPE (10 + 10 μg μl^–1^): 15.44 ± 7.62, *p* < 0.05; and control: 61.78 ± 10.57 vs. MLE + PPE (20 + 20 μg μl^–1^): 7.93 ± 1.12, *p* < 0.001] (Figure 5A4). Similar results were obtained for *S. oralis* [control: 33.02 ± 8.2 vs. MLE + PPE (10 + 10 μg μl^–1^): 7.01 ± 4.16, *p* < 0.05; and control: 33.02 ± 8.2 vs. MLE + PPE (20 + 20 μg μl^–1^): 1.68 ± 1.19, *p* < 0.05] (Figure 5B4); *S. mitis* [control: 23.78 ± 6.45 vs. MLE + PPE (10 + 10 μg μl^–1^): 5.06 ± 2.24, *p* < 0.05; and control: 23.78 ± 6.45 vs. MLE + PPE (20 + 20 μg μl^–11^): 2.18 ± 0.17, *p* < 0.05] (Figure 5C4); *R. dentocariosa* [control: 52.33 ± 12.13 vs. MLE + PPE (10 + 10 μg μl^–1^): 12.49 ± 4.75, *p* < 0.05; and control: 52.33 ± 12.13 vs. MLE + PPE (20 + 20 μg μl^–1^): 3.9 ± 0.48, *p* < 0.001] (Figure 5D4); and also the mixture [control: 68.2 ± 8.71 vs. MLE + PPE (10 + 100 μg μl^–1^): 22.26 ± 6.92, *p* < 0.001; and control: 68.2 ± 8.71 vs. MLE + PPE (20 + 20 μg μl^–1^): 19.25 ± 1.58, *p* < 0.001] (Figure 5E4).

In addition, the morphology of biofilms on treated glass appeared altered with respect to control, with more intense fluorescent signals. This suggests the presence of dead microbial cells with compromised membranes: PI can easily cross them and intercalate in greater quantity into double-stranded nucleic acids by emitting a clearer red signal (Rosenberg et al., 2019).

## Discussion

In a previous work, the authors demonstrated that hydroalcoholic polyphenolic extracts of *M. communis* L. leaf and *P. granatum* L. fruit peel showed the ability to inhibit the growth and survival of cariogenic bacteria of dental plaque (Sateriale et al., 2020). Moreover, their remarkable synergistic effects, also in association with amoxicillin, have been demonstrated (Sateriale et al., 2020). The characterization of the hydroalcoholic pomegranate peel extract showed that the most abundant phenolic compounds include pedunculagin 1, punicalin, ellagic acid hexose, ellagic acid pentose, and ellagic acid deoxesose (Pagliarulo et al., 2016), while gallic acid derivatives, tannins, myricetin, and quercetin derivatives are the most abundant phenolic compounds in hydroalcoholic extracts prepared from myrtle leaf (Sateriale et al., 2020). These results allowed us to design the goals of this study and to evaluate the antibiofilm activity of the same natural extracts in order to encourage their use as alternative options to prevent and treat oral infectious diseases, including biofilm-related ones.

Since adherence on oral surfaces is the first crucial step in biofilm formation in the oral cavity (Lendenmann et al., 2000), before analyzing the antibiofilm effects of myrtle and pomegranate extracts against dental plaque bacteria, the first objective of this work was to determine the ability of *S. mutans* ATCC 25175, *S. oralis* SO1, *S. mitis* SM2, and *R. dentocariosa* RD1 oral isolates to adhere and then to form biofilms. The results obtained by TM and TCPM methods showed the ability to form biofilm both on polystyrene tube walls and on polypropylene surface of 96-well microtiter plates, for all tested oral isolates. No oral isolates were indeed found to be negative for the TM test. In particular, *S. mutans* strain was shown to be strongly positive (+ + +), as well as the multispecies biofilm, while *S. oralis* and *R. dentocariosa* isolates were moderately positive (+ +), and finally the *S. mitis* isolate was weakly positive (+) (Supplementary Table S1). These results were confirmed by the quantification of microbial biofilm biomass through the recording of the OD (Table 1) at 600 nm by a microtiter reader. Interestingly, the high OD values measured for the *S. mutans* single biofilm (mean OD_600 *nm*_ of 0.506 ± 0.045) and for the multispecies biofilm (mean OD_600 *nm*_ of 0.607 ± 0.028) allow their classification as strongly adherent microbial isolates. *S. oralis* and *R. dentocariosa* isolates were moderately adherent, while the *S. mitis* isolate was weakly adherent. Both TM and TCPM proved to be effective methods to assess *in vitro* microbial biofilm formation by oral pathogens. However, given several disadvantages related to TM, in terms of major quantity of needed material for each sample and minor number of samples to test simultaneously, TCPM was preferred for subsequent assays. Crystal violet staining is a commonly used technique to quantify biofilm formation, thanks to its relatively easy execution, its reproducibility, and its ability to rapidly analyze multiple samples simultaneously (Stepanović et al., 2007; Agarwal et al., 2011; Liu et al., 2018). Considering these advantages and the interconnection between EPS production and microbial biomass amount in biofilms (Yallop et al., 2000), this staining has been selected as an adequate method to assess biofilm formation in the absence and in the presence of the natural extracts tested in this study. The first 12 h of biofilm formation was shown to correspond to the first adhesion stage, as indicated by the observed increase of OD values. With the increase in cell proliferation and the generation of extracellular matrix, the exponential growth was reached, coming to the plateau (or stationary phase) at about 18 h of monitoring, up to 48 h. No oral isolates showed significant differences in biofilm formation amount between 24 and 48 h of growth. These results, in accordance with other studies (Servetas et al., 2016; Mira et al., 2019), allow us to confirm that 24 h are enough to observe a stable biofilm growth by *S. mutans* ATCC 25175, *S. oralis* SO1, *S. mitis* SM2, and *R. dentocariosa* RD1 oral isolates and by their multispecies biofilm.

Subsequently, the antibiofilm activity of polyphenolic extracts against *S. mutans* ATCC 25175, *S. oralis* SO1, *S. mitis* SM2, and *R. dentocariosa* RD1 oral isolates was measured. Both 50% hydroethanolic myrtle leaf polyphenolic extracts (MLE) and pomegranate peel polyphenolic extracts (PPE) were able to inhibit the formation of biofilms made by all mentioned oral isolates, as well as to disrupt preformed 1-day biofilms. Interestingly, the binary combination of myrtle and pomegranate extracts (MLE + PPE) inhibited the biofilm formation and eradicated mature biofilm at lower concentrations with respect to the extracts individually tested.

These results suggest that the antibiofilm activity of polyphenolic myrtle leaf and pomegranate peel extracts, especially in binary combination, against *S. mutans* ATCC 25175, *S. oralis* SO1, *S. mitis* SM2, and *R. dentocariosa* RD1 oral isolates is in line with other studies that demonstrated the ability of *P. granatum* L. fruit peel and *M. communis* L. leaf extracts to inhibit the biofilm formation process and to disrupt preformed biofilms (Bakkiyaraj et al., 2013; Mansouri et al., 2013). The potential of the tested natural extracts in our study to prevent microbial adhesion could be closely linked to the antibiofilm properties of the extracted polyphenolic compounds. Interestingly, flavanols and flavonoids, such as rutin from myrtle extract, exhibited strong sortase inhibitory activity, thus interfering with the aptitude of bacteria to adhere to surfaces (Kang et al., 2006). In general, among the mechanisms underlying the antibacterial activity of polyphenols are the damage to the cytoplasmic membrane and the inhibition of nucleic acids and of energy metabolism but also the inhibition of cell membrane synthesis and cell wall synthesis (Cushnie and Lamb, 2011). As for the mechanisms behind the antibiofilm effects of polyphenols, although they are still unclear, they could be attributable to multiple factors acting in concert. The adhesion process to the surface can be influenced mainly by the physicochemical properties of the surface, but also by the bacterial characteristics and by environmental factors. The alteration of the bacterial surface could interfere with the normal cell–substrate interactions and compromise the attack phase and the normal biofilm development process (Simões et al., 2007). The researches on plant polyphenol antibiofilm activity have revealed several activities leading to biofilm suppression, for example, by affecting the bacterial regulatory mechanisms, such as quorum sensing (Silva et al., 2016) or by damaging the stability of the bacterial cytoplasmic membrane after inhibition of lipid metabolism (Rozalski et al., 2013). In addition, polyphenols could exert an aggregatory effect on bacterial cells, thus determining a preferred interaction of bacterial cells between themselves rather than with the surface (Cushnie et al., 2007). This could explain the ability of polyphenolic extracts to eradicate preformed biofilms, but future studies are needed to clarify their mechanisms of action. Regarding the synergistic antibiofilm effects of myrtle and pomegranate extracts, they could depend on their different polyphenolic contents and relative mechanisms of action. In particular, recent literature data showed that flavonoids, such as quercetin of myrtle extract, are able to inhibit the activities of glucosyltransferases and F-ATPase, and the acid production by *S. mutans* cells, thus significantly affecting biofilm development and acidogenicity (Duarte et al., 2006), while gallic acid and tannins showed suppressive effect on *S. mutans* biofilm formation by inhibition of glucosyltransferase and fructosyltransferase (Sendamangalam et al., 2011). On the other hand, the mechanisms of action linked to antibiofilm activity of pomegranate polyphenolic extracts implicate both the precipitation of vital proteins involved in the formation of biofilms, such as adhesins, and the alteration of the cell surface charge thereby interfering with cell–substratum interactions and biofilm development, mediated by ellagic acid and ellagitannins, respectively (Liu et al., 2008).

In addition, to our knowledge, several studies demonstrated that pomegranate peel extracts do not exert cytotoxic effects on human fibroblast (Eid et al., 2015) and that could interfere with gingival fibroblast viability only at high concentrations (Celiksoy et al., 2020). Moreover, literature data reported that phytocompounds from *M. communis* L. leaves induce apoptosis only in cancer cell lines, but not in non-transformed human fibroblasts (Asgarpanah and Ariamanesh, 2015). In addition, the absence of cytotoxicity of myrtle derivatives is confirmed also by studies in humans. For example, the use myrtle paste was shown to be effective for increasing the quality of life of patients who suffer from painful oral condition, such as aphthous stomatitis, without toxic effects on mouth epithelial (Babaee et al., 2010). However, further research, including cytotoxicity assays and *in vivo* studies, is required to reach the practical application of these natural antimicrobials in clinical use to counteract oral biofilms.

In conclusion, this study opens toward new perspectives in using natural antimicrobial combined therapies against oral pathogens able to form biofilm. Although only some of the bacterial species forming the complex oral plaque community have been taken into consideration in this study, our data encourage the promotion of clinical trials for the development of innovative natural tools in the prophylaxis and treatment of biofilm-related oral diseases.

## Data Availability Statement

The raw data supporting the conclusions of this article will be made available by the authors, without undue reservation.

## Author Contributions

DS and CaP conceived the work. RI contributed to microscopy analyzes. RC, ChP, EV, MV, PS, and MP were involved in the data analysis, wrote and critically reviewed the manuscript. All authors read and approved the final manuscript.

## Conflict of Interest

PS was employed by CEINGE. The remaining authors declare that the research was conducted in the absence of any commercial or financial relationships that could be construed as a potential conflict of interest.
