# Supplementary material for: Phytocompounds vs. Dental Plaque Bacteria: In vitro Effects of Myrtle and Pomegranate Polyphenolic Extracts Against Single-Species and Multispecies Oral Biofilms
Source: Front Microbiol. 2020 Nov 5;11:592265. doi: 10.3389/fmicb.2020.592265 (PMC7674652; doi:10.3389/fmicb.2020.592265)
Supplement: Supplementary file 1 [file Table_1.DOCX]

**Table S1. Biofilms adherence levels of *S. mutans* ATCC 25175, *S. oralis* SO1, *S. mitis* SM2 and *R. dentocariosa* RD1 oral isolates, in pure and mixed colture, according Tube Metod (TM).**

| **Bacterial isolate/Control** | **Bacterial adherence level** | | |
| --- | --- | --- | --- |
| **Control** (Brain Hearth Infusion broth) | negative | 0 | **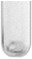** |
| ***Streptococcus mutans* ATCC 25175** | strongly positive | **+ + +** | 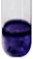 |
| ***Streptococcus oralis* SO1** | moderately positive | **+ +** | 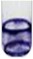 |
| ***Streptococcus mitis* SM2** | weakly positive | **+** | 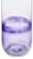 |
| ***Rothia dentocariosa* RD1** | moderately positive | **+ +** | 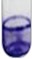 |
| **Mixed culture** (1:1:1:1 ratio) | strongly positive | **+ + +** | 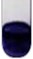 |
